# Sandy Everlasting (*Helichrysum arenarium* (L.) Moench): Botanical, Chemical and Biological Properties

**DOI:** 10.3389/fpls.2018.01123

**Published:** 2018-08-07

**Authors:** Dejan Pljevljakušić, Dubravka Bigović, Teodora Janković, Slavica Jelačić, Katarina Šavikin

**Affiliations:** ^1^Institute for Medicinal Plant Research “Dr Josif Pančić”, Belgrade, Serbia; ^2^Faculty of Agriculture, University of Belgrade, Belgrade, Serbia

**Keywords:** *Helichrysi flos*, sandy, everlasting, immortelle, chemistry, cholagogue, naringenin

## Abstract

Sandy everlasting [*Helichrysum arenarium* (L.) Moench] is herbaceous perennial plant belonging to Asteraceae family and it is native to Europe, Central Asia, and China. It belongs to the section HELICHRYSUM (Asteraceae family, genus *Helichrysum*) along with *H. plicatum* DC. Prodr., which very similar phenolic profile and *H. italicum* (Roth), which is widely used for essential oil extraction. Its flowers have a long tradition in European ethnomedicine as a cholagogue, choleretic, hepatoprotective, and detoxifying herbal drug. The flowers are rich in phenolic compounds including flavonoids, chalcones, phenolic acids, coumarins, and pyrones. Apart from polyphenols, other compounds such as sterols, lignans, and glycosides of aromatic compounds have been also isolated from *H. arenarium*. The majority of authors confirm that the most important group of compounds responsible for biological activities is flavonoids. Moreover, significant activities of naringenin, one of the main flavonoids of *H. arenarium*, were reported. On the other hand, there are no clinical data about testing the extracts or preparations based on *H. arenarium*. Although *H. arenarium* is well known in phytotherapy for its potential in the treatment of gallbladder disease and are classified as endangered species in a number of European countries, very few data about its cultivation are available in the literature.

## Introduction

Sandy everlasting [*Helichrysum arenarium* (L.) Moench] has a long tradition in European ethnomedicine as a medicinal plant which is attributed to cholagogue, choleretic, hepatoprotective, and detoxifying activities (Czinner et al., 2001). It is herbaceous perennial plant belonging to Asteraceae family and it is native to Europe, Central Asia, and China (Anderberg and Anderberg, 2005; Erhardt et al., 2008; Yang et al., 2009). The origin of the genus name is derived from the Greek words *helios*, meaning sun, and *chrysos*, meaning gold, what refers to the shiny-golden color of inflorescence (Maznev, 2004). The main biologically active compounds of *Helichrysi arenarii inflorescentia* (syn. *H. a. flos*) are flavonoids, with chalcone isosalipurposide, and flavanones salipurposide, prunin and naringenin as dominant constituents, while other compounds present in remarkable amount are phtalides, carotenoids, essential oil and yellow pigments: α-pyron derivates such as arenol and homoarenol (Bryksa-Godzisz et al., 2006; Kurkina et al., 2012). Nevertheless, Smirnova and Pervykh (1998) reported that it is the sum of flavonoids that account for the cholagogue activity of the everlasting flower extracts.

Although *Helichrysi arenarii flos* has not been included in the European Pharmacopoeia, the State Pharmacopoeia of the USSR, Pharmacopoeia Helvetica and Polish Pharmacopoeia listed it as officinal drug (Ph. Helv. VII, 1987; Ph. USSR, 1990; Polish Ph VI, 2002; Ph. Eur. 7.0., 2011) for its choleretic and cholagogue activity. Furthermore, due to the long usage in traditional medicine as well as proven therapeutic properties, it has been included in the monographs of the World Health Organization, German Commission E, Physician's desk reference for herbal medicines and German pharmaceutical codex (Blumenthal et al., 1998; PDR, 1998; DAC, 2005; WHO, 2015).

Synthetic drugs which are used in the treatment of gallbladder, dyspeptic, and liver disorders are often inadequate and may sometimes lead to serious side-effects. Medicinal plants could be a fundamental source of potentially useful new compounds for the development of effective cure to fight a variety of gastroenterology problems. According to the WHO monographs (WHO, 2015), *Helichrysi flos* (biological source *Helichrysum arenarium* (L.) Moench) is used as a cholagogue, in the treatment of dyspeptic diseases, and in traditional medicine as choleretic, diuretic, mild spasmolytic, hepatoprotective agent, and for detoxification.

The main suppliers of sandy everlastings' flower heads on market are former USSR countries, Poland and Turkey (Bisset and Wichtl, 1994; WHO, 2015). There are three forms of the drug in the trade: dry flower heads (*Helichrysi arenarii flos*), fluid extract *(Helichrysi arenarii extractum fluium)*, usually obtained by extraction with water-ethanol or glycerin and dry extract of flower heads (*Extractum florum Helichrysi arenarii siccum*), usually obtained from fluid extract (EMA, 2015). The flower heads are mainly used for decoctions, while dry extracts are widely used for the production of galenic preparations in the form of capsules and tablets. These forms on the market are mainly represented by Russian preparations where the most popular is “Flamin tablets” (Kurkina et al., 2012).

*H. arenarium* is fully protected in Sweden and Serbia, while in Denmark and Estonia the species is listed as “care demanding” (Lilleleht, 1998; Butorac, 1999; Olsson et al., 2005). In the 1970s the species received partial legal protection in Poland, which stopped the overexploitation of its natural resources (Sawilska and Jendrzejczak, 2013). Besides the unsustainable collection, the main risk factor that threatens species subsistence is the transformation of natural habitat to agricultural land (orchards, vineyards) and areas for the cultivation of fast-growing tree species (varieties of poplar, pine, and acacia) (Butorac, 1999). Protection regulations triggered a need to develop a new efficient growing technology for sandy everlasting, which could restore the species for it use in phytotherapy (Sawilska and Jendrzejczak, 2013). After first unsuccessful attempts of growing sandy everlasting in plantations, Sawilska et al. (2009) have explained the reason why those attempts failed and pointed out the influence of mycorrhizal fungi on the inflorescence yield. Small farms in Latvia also cultivate *H. arenarium* for local consumption, where the production area was estimated as less than 2 ha since marketing problems have affected the production of domestic medicinal plants in general (Olsson et al., 2005).

This survey aims to systematize the published knowledge about sandy everlasting so far and to highlight the importance of knowledge of botanical, chemical and pharmacological properties of this herbal drug, together with overview of cultivation approaches.

## Botanical description, taxonomy, and distribution

Sandy everlasting is hardy perennial with a deep growing root system (Olsson et al., 2005). The plant grows 10–30 (50) cm high with obliquely descendent, strong and short rhizome (Butorac, 1999; WHO, 2015). The stem is usually branched at the upper part and carries alternate leaves, which are 2–5 cm in length (Olsson et al., 2005). The rosette leaves are reverse ovoid, while upper leaves are linear-lanceolate (Butorac, 1999). Both the leaves and the stem are covered with gray or silvery wooly hairs (Olsson et al., 2005). The inflorescence is capitula, numerous, globose, 3–6 (9) mm in diameter and 10–30 (100) capitulas are grouped in false umbels (WHO, 2015). Phyllaries ca. 50, slightly loosely arranged in 3–7 rows, often with the declined tip at end of anthesis, bright lemon-yellow, more pallid yellow, pinkish, or orange; outer ones obovate or elliptic, abaxially densely villous, apex rounded; inner ones widely oblong-spatulate to sublinear. Florets (25–50) are almost always hermaphrodite, tubular-infundibulate, sometimes marginal florets are only female, pappus of about 30 yellowish-white hairs, as long as the corolla; pollinated by insects (Gajić, 1975; Yousheng et al., 2011). Fruit is pentagonal, oblong, brown achene, 0.7–1.2 mm long, with a pappus (WHO, 2015).

Sandy everlasting (*H. arenarium* (L.) Moench) belongs to the section HELICHRYSUM (Asteraceae family, genus *Helichrysum*) along with *H. plicatum* DC. Prodr., which has according to Bigović et al. (2011) very similar phenolic profile and *H. italicum* (Roth), which is widely used for essential oil extraction. Botanical division of European taxa in genus *Helichrysum* Mill. is listed in Table 1.

**Table 1:** Botanical division of European taxa in genus *Helichrysum* Mill. (Flora Europea, 2006).

**#**	**Genus - *Helichrysum* Mill**.
Sect. VIRGINEA (DC) Fiori		
1		*H. amorginum* Boiss. and Oprh.
2		*H. sibthorpii* Rouy
3		*H. doerfleri* Rech.fil
4		*H. frigidum* (Labill.) Wild.
Sect. HELICHRYSUM		
5		H. stoechas^*^
6		*H. rupestre* (Rafin.) DC.^*^
7		*H. heldreichii* Boiss.^*^
8		*H. ambiguum* (Pers)^*^
9		H. saxatile^*^
10		*H. italicum* (Roth)^*^
11		*H. orientale* (L.)
12		*H. plicatum* DC. Prodr.
13		*H. arenarium* (L.) Moench
14		*H. graveolens* (Bieb.)
Sect. XEROCHLEANA (DC.) Bentham		
15		*H. foetidum* (L.) Cass
16		*H. bracteatum* (Vent.) Andrews

* *These species are classified as woody perennials in H. stoechas group*.

The Global Biodiversity Information Facility has reported five *H. arenarium* subspecies with several synonyms listed in Table 2. On the other hand, Galbany-Casals et al. (2009) recognized only two subspecies *arenarium* and *aucheri*. Nevertheless, according to EMA (2015), most of *Helichrysi flos* from EU countries belong to *H. arenarium* subsp. *arenarium* subspecies.

**Table 2:** List of *Helichrysum arenarium* subspecies (GBIF, 2013).

**Scientific name**	**Synonym**
*Helichrysum arenarium* (L.) Moench	*Helichrysum arenarium* (L.) Moench subsp. *arenarium*
	*Gnaphalium arenarium* L.
	*Gnaphalium adscendens* Thunb.
	*Gnaphalium aureum* Gilib.
	*Gnaphalium buchtormense* Sch.Bip.
	*Gnaphalium elichrysum* Pall.
	*Cyttarium arenarium* Peterm.
	*Stoechas citrina* Gueldenst.
*Helichrysum aucheri* Boiss.	*Helichrysum arenarium* subsp. *aucheri* (Boiss.) P. H. Davis and Kupicha
	*Helichrysum arenarium* subsp. *erzincanicum* P. H. Davis and Kupicha
*Helichrysum corymbiforme* Katina	*Helichrysum arenarium* Moench subsp. *ponticum* (Velen.) Clapham
*Helichrysum thianschanicum* Regel	*Helichrysum arenarium* var. *kokanicum* Regel and Schmalh.
	*Helichrysum kokanicum* (Regel and Schmalh.) Krasch. and Gontsch.
	*Helichrysum thianschanicum* var. *aureum* O.Fedtsch. and B.Fedtsch.
*Helichrysum rubicundum* (K. Koch) Bornm.	*Helichrysum arenarium* subsp. *rubicundum* (K.Koch) P.H.Davis and Kupicha

**Figure 1:**
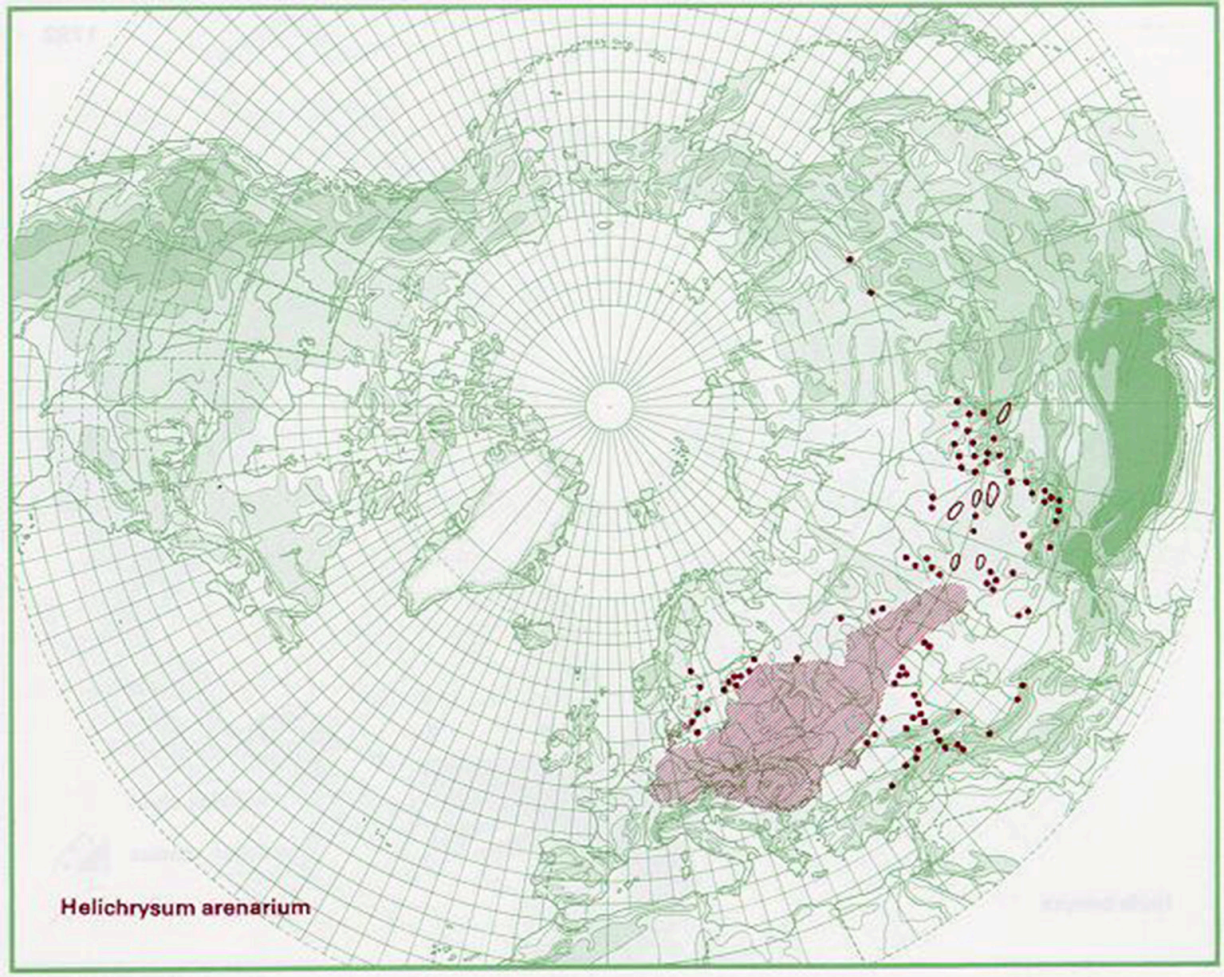
Distribution map for *Helichrysum arenarium*. Source: The virtual flora. Naturhistoriska Riksmuseet, Sweden, with permission of Anderberg and Anderberg (2005).

This species is broadly distributed in Europe, western Siberia, and central Asia (Kirpičnikov, 1959). Grows on dry sandy places, from Netherlands, Sweden, and Estonia, southwards to Germany, Bulgaria, and Kazakhstan (EMA, 2015). According to Euro+Med Plantbase reports the occurrence of *H. arenarium* is present from the Bay of Biscay to Ural mountain and from southern Scandinavia to the northern parts of Balkan peninsula (Greuter, 2006). Distribution of the species is also proposed graphically by Anderberg and Anderberg (2005) in Figure 1. In Serbia, *H. arenarium* area is limited only to two sandy sites Kladovska and Deliblatska peščara (Sarić, 1989).

## Traditional uses

*Helichrysi flos* (biological source *H. arenarium*) is well-known species in traditional medicine. It is used as a choleretic, hepatoprotective and detoxifying agent, diuretic, as a mild antimicrobial and spasmolytic agent (Cosar and Cubukcu, 1990; Czinner et al., 2000, 2001; Bigović et al., 2010, 2011). The flowers contain antibacterial constituents and bitter substances, which may also promote gastric and pancreatic secretion (Amirdowlat Amasyaci, 1990). It is also indicated for indigestion as well as for loss of appetite (Turova and Sapozhnikova, 1984). The average daily dosage is 3 g of the drug or equivalent preparations (Blumenthal et al., 1998; WHO, 2015).

In Russia, the inflorescence of *H. arenarium* has been applied in the form of infusions for stimulating gastric secretion, treating of gallbladder disorders as well as cystitis, rheumatism, arthritis, and gout (Shikov et al., 2014). Moreover, *Flores Helichrysi arenarii* is included in USSR Ph. USSR (1990). Recommended administration of decoction is 1:20, 100 mL 2–3 times per day. Furthermore, tablets “Flamin” containing purified flavonoids and studies showed that dose of 50 mg taken 3 times/day in a period of 40 days was safe (Sokolov, 2000).

In Serbian traditional medicine, galenic preparations with *Helichrysi flos* are prepared to diminish the concentration of bile acids, increase the content of bilirubin in bile and tonus of gallbladder and to promote the secretion of bile. Moreover, a spasmolytic effect on the sphincter of the gallbladder is stated. It is used in the form of tea as infusion or decoction (Tasić et al., 2009). According to European Medicinal Agency (EMA) in Austria *Helichrysi flos* is used in a form of tea mixtures prepared in pharmacies. Also, *H. arenarium* inflorescence was introduced to the official medicine in Poland, during the thirties of the XX century. Currently, on the Polish market there are two products present in a category of “Pharmacopoeial products.” Outside the EU countries *Helichrysi flos* preparations have been used first in Soviet Union and in countries of the former USSR. Kažemekaitis (2010) has compiled the historical data on the use of medicinal plant species in Lithuania since 1873 and he reported that *H. arenarium* was mentioned in the official regulations in 1904, 1911, 1914, and during USSR times. Until now, there are no clinical data about testing the preparations based on *H. arenarium*.

## Chemical constituents

The flowers of sandy everlasting (*H. arenarium*) are the rich source of phenolic compounds including flavonoids, chalcones, phenolic acids, phthalides, coumarins, and pyrones. Apart from polyphenols, other compounds such as sterols, lignans, and glycosides of aromatic compounds have been also isolated and identified from *H. arenarium*. Table 3 summarizes the chemical constituents that have been reported in the literature to date.

**Table 3:** Reported chemical compounds in sandy everlating (*Helichrysum arenarium*).

**Classification**	**No**.	**Compound name**	**Reference**
Chalcones	1	chalconaringenin-2'-*O*–β-D-glucoside (isosalipurposide)	Hänsel et al., 1960; Bryksa-Godzisz et al., 2006; Morikawa et al., 2009a; Kurkina et al., 2012; Jarzycka et al., 2013
	2	chalconaringenin 2',4'-di-*O*–β-D-glucoside	Morikawa et al., 2009a
	3	chalconaringenin 2'-*O*–β-D-diglucoside (arenariumoside III)	Morikawa et al., 2009a
Flavanones	4	naringenin	Vrkoc et al., 1973; Czinner et al., 2000; Bryksa-Godzisz et al., 2006; Eshbakova and Aisa, 2009; Albayrak et al., 2010; Jarzycka et al., 2013
	5	(2R)-naringenin-5-*O*-glucoside (helichrysin A)	Hansel and Heise, 1959; Morikawa et al., 2009a
	6	(2S)-naringenin-5-*O*-glucoside (helichrysin B (= salipurposide))	Hansel and Heise, 1959; Morikawa et al., 2009a; Kurkina et al., 2012
	7	(2S)-naringenin-7-*O*–β-D-glucoside	Morikawa et al., 2009a; Yang et al., 2009;
	8	(2S)-naringenin-5,7-di–*O–*β-D-glucoside	Morikawa et al., 2009a
	9	(2R)-naringenin-5,7-di–*O–*β-D-glucoside	Morikawa et al., 2009a
	10	(2S)-naringenin-5–*O–*diglucoside (arenariumoside I)	Morikawa et al., 2009a
	11	(2R)-naringenin-5–*O–*diglucoside (arenariumoside II)	Morikawa et al., 2009a
	12	(2S)-naringenin-5,4'-di–*O–*glucoside (arenariumoside IV)	Morikawa et al., 2009a
	13	(2R)-aromadendrin-5–*O–*glucoside (helicioside A)	Morikawa et al., 2009a
	14	(2R)-eriodictyol-5–*O–*β-D-glucoside	Wang et al., 2009
	15	(2S)-5,8,4'-trihydroxy-6,7-vinylenedioxyflavanone	Yong et al., 2011
Flavonols	16	kaempferol	Czinner et al., 2000; Sroka et al., 2004; Bryksa-Godzisz et al., 2006;
	17	kaempferol-3-O–β-D-glucoside (astragalin)	Czinner et al., 2000; Sroka et al., 2004; Morikawa et al., 2009a; Yang et al., 2009; Jarzycka et al., 2013
	18	kaempferol-3–*O–*gentiobioside	Morikawa et al., 2009a
	19	kaempferol-3–*O–*laminaribioside	Morikawa et al., 2009a
	20	kaempferol 3,7-di–*O*–β-D-glucoside	Morikawa et al., 2009a
	21	kaempferol 3,4'-di–*O*–β-D-glucoside	Morikawa et al., 2009a
	22	(2R,3R)-dihydrokaempferol-7–*O–*β-D-glucoside	Morikawa et al., 2009a
	23	quercetin	Czinner et al., 2000; Sroka et al., 2004; Bryksa-Godzisz et al., 2006
	24	quercetin-3-*O*–β-D-glucoside (isoquercitrin)	Czinner et al., 2000; Bryksa-Godzisz et al., 2006; Morikawa et al., 2009a; Yang et al., 2009; Jarzycka et al., 2013
	25	quercetin-3,3'-di-O–β-D-glucoside	Morikawa et al., 2009a
	26	quercetin-3–*O–*rutinoside (rutin)	Morikawa et al., 2009a
	27	3,5-dihydroxy-6,7,8-trimethoxyflavonol	Vrkoc et al., 1973; Yong et al., 2011
Flavones	28	luteolin	Smirnova and Pervykh, 1998; Czinner et al., 2000; Bryksa-Godzisz et al., 2006; Albayrak et al., 2010
	29	luteolin 7-O–β-D-glucoside	Czinner et al., 2000; Morikawa et al., 2009a
	30	luteolin-3'-O–β-D-glucoside	Morikawa et al., 2009a
	31	6-hydroxyluteolin 7-O–β-D-glucoside	Morikawa et al., 2009a
	32	apigenin	Czinner et al., 2000; Sroka et al., 2004; Bryksa-Godzisz et al., 2006; Albayrak et al., 2010
	33	apigenin-7-O–β-D-glucoside	Czinner et al., 2000; Sroka et al., 2004; Bryksa-Godzisz et al., 2006; Morikawa et al., 2009a; Albayrak et al., 2010; Jarzycka et al., 2013
	34	apigenien-7-O–β-D-glucosiduronic acid methyl ester	Morikawa et al., 2009a
	35	apigenin-7–*O–*gentiobioside	Morikawa et al., 2009a
	36	apigenin-7,4'-di-O–β-D-glucoside	Morikawa et al., 2009a
	37	scutellarein-7–*O–*gentiobioside	Morikawa et al., 2009a
	38	diosmetin-7–*O–*rutinoside (diosmin)	Eshbakova and Aisa, 2009
	39	5,6,4'-trihydroxy-3'-methoxyflavone 7-*O*–β-glucoside	Morikawa et al., 2009a
Phenolic acids	40	chlorogenic acid	Czinner et al., 2000; Bryksa-Godzisz et al., 2006; Albayrak et al., 2010; Jarzycka et al., 2013
	41	caffeic acid	Dombrowicz et al., 1992; Czinner et al., 2000; Sroka et al., 2004; Albayrak et al., 2010
	42	*p*-cumarinic acid	Dombrowicz et al., 1992; Sroka et al., 2004; Albayrak et al., 2010
	43	ferulic acid	Dombrowicz et al., 1992; Bryksa-Godzisz et al., 2006
	44	sinapic acid	Dombrowicz et al., 1992
	45	3,4-methylendioxycinnamic acid	Yong et al., 2011
	46	syringic acid	Dombrowicz et al., 1992; Sroka et al., 2004
	47	protocatechiuc acid	Dombrowicz et al., 1992; Sroka et al., 2004
	48	vanillic acid	Dombrowicz et al., 1992
	49	*p*-hydroxybenzoic acid	Dombrowicz et al., 1992; Sroka et al., 2004
	50	gentisic acid	Dombrowicz et al., 1992
α-pyranones	51	arenol	Hänsel et al., 1960; Vrkoč et al., 1971
	52	homoarenol	Hänsel et al., 1960; Vrkoč et al., 1971
Phthalides	53	5,7-dihydroxyphthalide	Vrkoc et al., 1973; Kurkina et al., 2012
	54	5-methoxy-7-hydroxyphthalide	Vrkoc et al., 1973; Kurkina et al., 2012
	55	5-methoxy-7-O–β-D-glucosyl phtalide	Morikawa et al., 2009a
	56	helichrysumphtalide	Eshbakova and Aisa, 2009
	57	everlastoside H	Morikawa et al., 2009b
Coumarines	58	umbelliferone	Derkach et al., 1986
	59	scopoletin	Derkach et al., 1986
	60	scopoletin 7-glucoside (scopolin)	Morikawa et al., 2009a
Sterols	61	β-sitosterol	Eshbakova and Aisa, 2009; Yong et al., 2011
	62	β-sitosterol–β-D-glucoside	Eshbakova and Aisa, 2009; Yong et al., 2011
	63	stigmasterol	Yong et al., 2011
	64	stigmasterol–β-D-glucoside	Yong et al., 2011
Other compounds	65	2-hydroxy-4,6–*O–*D-glucosyloxy-benzoic acid	Morikawa et al., 2009a
	66	benzyl–β-primeveroside	Morikawa et al., 2009a
	67	icariside F_2_	Morikawa et al., 2009a
	68	benzoyl-O–β-gentiobioside	Morikawa et al., 2009a
	69	2-phenylethyl–β-primeveroside	Morikawa et al., 2009a
	70	2-phenylethyl–β-gentiobioside	Morikawa et al., 2009a
	71	icariside D_1_	Morikawa et al., 2009a
	72	syringin	Morikawa et al., 2009a
	73	dihydrosyringin	Morikawa et al., 2009a
	74	eugenyl-O–β-glucopyranoside (citrusin C)	Morikawa et al., 2009a
	75	4-(3-glucopyranosyloxy-4-hydroxyphenyl)-(E)-3-buten-2-one	Morikawa et al., 2009a
	76	maltol-6'–*O–*β-apiofuranosyl-β-D-glucopyranoside	Morikawa et al., 2009a
	77	maltol-3–*O–*β-D-apiofuranosyl-β-D-glucopyranoside	Wang et al., 2012
	78	resveratrol	Albayrak et al., 2010
	79	aureusidin 6–*O–*β-D-glucoside	Morikawa et al., 2009a
	80	undulatoside A	Morikawa et al., 2009a
	81	tortoside B (manglieside E)	Morikawa et al., 2009a
	82	(7S,8R)-dihydrodehydrodiconiferyl alcohol-4-O–β-D-glucopyranoside	Morikawa et al., 2009a
	83	everlastoside E	Wang et al., 2009
	84-88	everlastoside I-M	Morikawa et al., 2009b
	89	orcinol–β-D-glucoside (sakakin)	Morikawa et al., 2009a
	90	licoagroside B	Morikawa et al., 2009b
	91	7–*O–*(β-glucopyranosyloxy)-5-hydroxy-1(3H)-isobenzofuranone	Morikawa et al., 2009b
	92	(E)-4-hydroxybenzalacetone-3–*O–*β-D-glucopyranoside	Wang et al., 2012
	93	4-allyl-2-methoxyphenyl-1–*O–*β-D-apiofuranosyl-(1-6)–*O–*β-D-glucopyranoside	Wang et al., 2012
	94	2,4,6-trihydroxylacetophenone-2,4-di–*O–*β-D-glucopyranoside	Wang et al., 2012
	95	everlastoside F	Morikawa et al., 2009b
	96	everlastoside G	Morikawa et al., 2009b
	97–100	everlastoside A-D	Wang et al., 2009
	101	oleanolic acid	Eshbakova and Aisa, 2009

### Flavonoids

**Figure 2:**
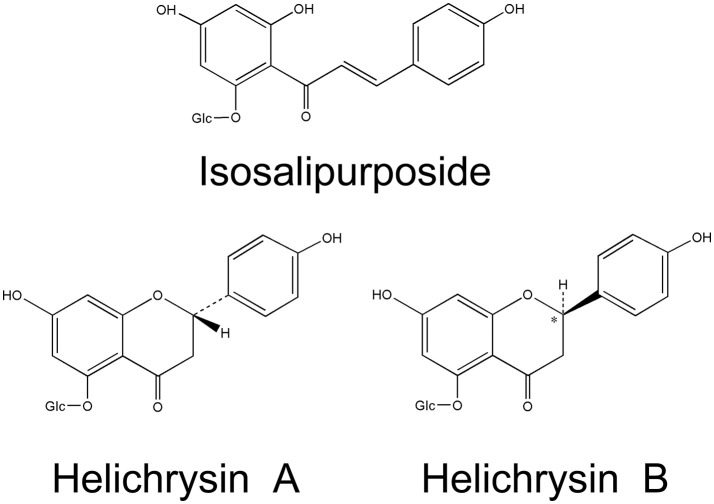
Chemical structures of three characteristic flavonoids originating from *H. arenarium* inflorescence [redrawn from WHO, 2015].

Flavonoids are the main characteristic components of *H. arenarium*, comprising 39 compounds. Chalcone isosalipurposide (**1**) and flavanones naringenin (**4**) and naringenin-5-*O*-glucoside are the dominant compounds in sandy everlasting (Czinner et al., 1999; Bryksa-Godzisz et al., 2006; Kurkina et al., 2012; Jarzycka et al., 2013). Hänsel and Heise (1959) reported two diastereomers of naringenin-5-*O*-glucoside, (+)-naringenin-5-β-D-glucoside (helichrysin A) (**5**) and (-)-naringenin-5-β-D-glucoside (helichrysin B) (**6**). It has been shown that helichrysin B is identical with salipurposide isolated from the bark of *Salix purpurea* (Charaux and Rabaté, 1931), and represent a racemic mixture of the naringenin-5-*O*-monoglucosides. Structural formulas of isosalipurposide, Helichirysin A, and Helichirysin A are presented in Figure 2. A number of naringenin glycosides have been also isolated (**7**–**12**), together with other flavanone glucosides (**13**–**15**). Flavone and flavonol compounds have been detected mainly as kaempferol (**16**–**22**), quercetin (**23**–**26**), luteolin (**28**–**31**), and apigenin (**32**–**37**) glycosides (Sroka et al., 2004; Bryksa-Godzisz et al., 2006; Morikawa et al., 2009a; Jarzycka et al., 2013). The major component is kaempferol-3-*O*-glucoside (**17**), followed by quercetin-3-*O*-glucoside (**24**), 6-hydroxyluteolin 7-*O*-glucoside (**31**) and apigenin-7-*O*-glucoside (**33**).

### Phenolic acids

Phenolic acids are also important class of compounds in the everlast inflorescence, and they are present as derivatives of hydroxycinnamic acid and hydroxybenzoic acid. Dombrowicz et al. (1992) have identified 11 phenolic acids by gas chromatography. Chlorogenic acid (**40**) is the main representative among hydroxycinnamic acid derivatives (Bryksa-Godzisz et al., 2006; Albayrak et al., 2010; Jarzycka et al., 2013). Caffeic (**41**), *p*-coumaric (**42**), and ferulic (**43**) acids are also typical compounds in *H. arenarium* (Dombrowicz et al., 1992; Sroka et al., 2004; Bryksa-Godzisz et al., 2006; Albayrak et al., 2010), while sinapic acid (**44**) and 3,4-methylendioxycinnamic acid (**45**) have been detected in minor amounts (Dombrowicz et al., 1992; Yong et al., 2011). Although the contents of these phenolic acids varied among different subspecies and populations, i.e., amount of chlorogenic acid is between 4.5 to 1,700 mg/100 g and caffeic acid amount is between 0.15 to 6.5 mg/100 g, their quantity in *Helichrysi flos* should not to be underestimated because they may contribute to the therapeutic effects of this medicinal raw material. Syringic acid (**46**) is the dominant hydroxybenzoic acid derivative (Dombrowicz et al., 1992; Sroka et al., 2004; Albayrak et al., 2010), followed by protocatechuic (**47**), vanillic (**48**) and *p*-hydroxybenzoic (**49**) acids, whereas gentisic acid (**50**) is present in the lowest amount.

### Phthalides, coumarins, and pyrones

**Figure 3:**
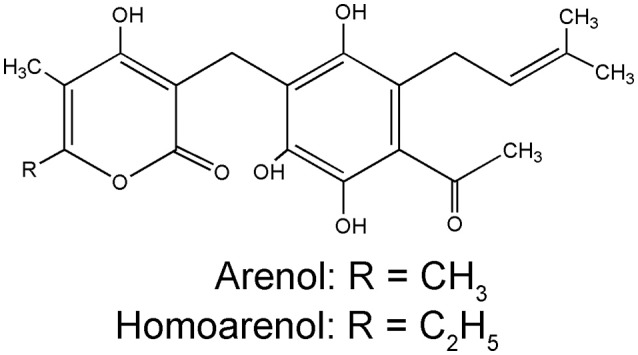
Chemical structures of two characteristic yellow pigments α-pyrons: arenol and homoarenol from the *H. arenarium* inflorescences [redrawn from Wichtl (2001)].

**Figure 4:**
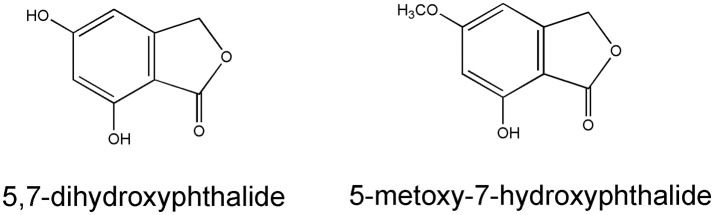
Chemical structures of two characteristic phthalides from the *H. arenarium* inflorescences [redrawn from Kurkina et al. (2012)].

Pyrone derivatives arenol (**51**) and homoarenol (**52**) have been isolated as yellow pigments (Vrkoč et al., 1971). Phthalides 5,7-dihydroxyphthalide (**53**) and 5-methoxy-7-hydroxyphthalide (**54**) are characteristic components of the *H. arenarium* inflorescence (Vrkoc et al., 1973; Kurkina et al., 2012), along with glycosides 5-methoxy-7-*O*-glucosyl phthalide (**55**), helichrysumphtalide (**56**), and everlastoside H (**57**) (Eshbakova and Aisa, 2009; Morikawa et al., 2009a,b). Coumarins umbelliferone (**58**), scopoletin (**59**) and its glucoside scopolin (**60**), have been detected in sandy everlasting flowers (Derkach et al., 1986; Morikawa et al., 2009a). Structures of pyrone derivatives arenol and homoarenol are presented in Figure 3, while major phthalides 5,7-dihydroxyphthalide and 5-methoxy-7-hydroxyphthalide are presented in Figure 4.

### Sterols

In the literature, 4 steroid compounds including β-sitosterol (**61**), β-sitosterol-glucoside (**62**), stigmasterol (**63**), and stigmasterol-glucoside (**64**) have been reported from *H. arenarium* (Eshbakova and Aisa, 2009; Yong et al., 2011).

### Other compounds

The largest group of other constituents detected in *H. arenarium* is glycosides of aromatic compounds. This includes phenolic acid glucoside (**65**), benzyl glycosides (**66**, **67**), benzyl ester glucoside (**68**), phenethyl glycosides (**69**-**71**), phenylpropanoid glucosides (**72**–**74**), phenylbutanoid glucoside (**75**), maltol glycosides (**76**, **77**) (Morikawa et al., 2009a; Wang et al., 2012). Most of the reported compounds were identified as monoglucosides containing glucose as sugar moiety. Diglycoside-compounds consist of glucose linked to glucose or other sugars such as xylose, rhamnose or apiose. Stilbene resveratrol (**78**) (Albayrak et al., 2010), aurone aureusidin 6-*O*-glucoside (**79**), chromone undulatoside A (**80**), lignan tortoside B (**81**), and neolignan glucoside (**82**) (Morikawa et al., 2009a) have been also identified in *H. arenarium*. Other aromatic glycosides (**83**–**94**) (Morikawa et al., 2009a,b; Wang et al., 2009, 2012) have been reported from sandy everlasting. Angeloyl glycosides (**95**, **96**) (Morikawa et al., 2009b) have been isolated, together with other glycosides (**97**–**100**) (Wang et al., 2009) and oleanolic acid (**101**) (Eshbakova and Aisa, 2009). Among other phenolic compounds, Jarzycka et al. (2013) also referred to catechins and proanthocyanidins.

### Essential oil composition

Chemical profiles of essential oils originating from different *Helichrysum* species, mainly *H. italicum*, but also *H. gymnocephalum, H. bracteiferum, H. selaginifolium, H. cordifolium, H. faradifani*, and *H. hypnoides* (Afoulous et al., 2011; Leonardi et al., 2013), has been investigated intensively by many researchers, while the number of investigations on the oil of *H. arenarium* plants is very limited (Judzentiene and Butkiene, 2006). Most of the papers dealing with sandy everlasting flowers' essential oil report content in an amount of about 0.04–0.09% (Cicin, 1962; Turova, 1974; Roth and Schmid, 1976; Czinner et al., 2000; Wichtl, 2001; Maznev, 2004). Data on chemical constituents presented in essential oils reported by various authors are very diverse.

Czinner et al. (2000) analyzed steam distilled essential oil from *H. arenarium* plants (0.09% yield) collected in the Caucasus region by GC and GC-MS where they identified 24 out of 60 compounds, which represent 83% of total oil. The most abundant group of compounds were aliphatic acids (34.6%) among which dodecanoic acid (11.9%), decanoic acid (9.8%) were the most dominant, followed by ester methyl palmitate (28.5%) and further aromatic compounds (10.2%) such as carvacrol and anethol (3.6 and 3.2%, respectively). On the other hand, Lemberkovics et al. (2001) reported, using the same analytical approach, that the most abundant compounds in the oils of Polish and Hungarian mercantile samples were methyl palmitate (28.5% and 21.7%, respectively), while caprinic acid (19.8%) was the main compound in oil of the cultivated plant's sample from Hungary. These discrepancies in chemical profiles could be consequence of different environmental factors such as insolation, soil type, precipitation level, etc. Furthermore, Judzentiene and Butkiene (2006) reported chemical profiles of essential oils from inflorescences and leaves of yellow and orange flowered sandy everlasting plants. Apparently, oils from inflorescences of both types of plants, yellow and orange, had two dominant components, β-caryophyllene and heneicosane, followed by α-copaene (9.0–25.6%, 3.0–32.1% and 1.5–7.2%, respectively). One of the main compound in the leaf essential oils in both plant types with yellow and orange inflorescences, besides β-caryophyllene, was δ-cadinene (9.8–22.3% and 6.6–11.8%, respectively), while other constituents presented in remarkable amount were 1,8-cineole, α-copaene, (E)-β-ionone, γ-cadinene, selina-3,7(11)-diene, epi-α-cadinol; α-cadinol, octadecane, isophytol, tricosane.

### Macro- and micro-elements

Very little attention has been paid to the content of macro- and microelements in *Helichrysi flos* herbal drugs and extracts. To the best of our knowledge, only one report done by Lemberkovics et al. (2002) partly studied this subject. Reported values of the observed elements in their highest levels were: Al (353 mg/kg), Cr (6 mg/kg), Cu (19 mg/kg), Mn (349 mg/kg), and P (2907 mg/kg) in the cultivated drug sample from Hungary, while the concentration of Ba (19 mg/kg), Ca (7575 mg/kg), Fe (159 mg/kg), and Zn (59 mg/kg) was highest in a commercial sample from Poland. Macro- and microelements are critical components for a number enzymatic and nonenzymatic processes involved in antioxidant defense of the human body, and a deficiency of any of these essential elements may impair the function of the overall antioxidant system (Zidenberg-Cherr and Keen, 1991).

## Pharmacological properties

The literature data related to the chemical profile of the *H. arenarium* inflorescences are very different, but the majority of authors confirms that the most important group of compounds responsible for biological activities are flavonoids, which can occur in aglycone and glycoside forms (Czinner et al., 1999, 2001, 2002; Wichtl, 2001; Lemberkovics et al., 2002; Olsson et al., 2005; Kurkina et al., 2012; WHO, 2015). Among flavonoid components in the inflorescence extracts, chalcone derivative isosalipurposide has been reported as the most abundant compound, and it has been indicated as responsible for the yellow color of the involucral bracts and for the hepatoprotective activity of the drug (Hänsel et al., 1960; Skakun and Stepanov, 1988; Czinner et al., 1999; Bryksa-Godzisz et al., 2006; Kurkina et al., 2012; Jarzycka et al., 2013; WHO, 2015).

According to the WHO (2015), the only use of *Helichrysi arenarii flos* described in pharmacopeias and well-established documents are the treatment of dyspeptic disorders. On the other hand, choleretic, cholagogue, hepatoprotective and detoxifying activity of the inflorescence of *H. arenarium* has been recognized for a long time in Europe (Kroeber, 1951; Szadowska, 1962; Wagner, 1993; Shikov et al., 2014). Furthermore, Szadowska (1962) has reported mild choleretic and spasmolytic effect of this drug observed on rats. In this research intravenous administration of three flavonoids from sandy everlasting (kaempferol-3-glycoside, naringenin-5-glycoside and apigenin) has been applied in a dose 4 mg/100 g vs. positive control (Decholin, deoxycholic acid) as well as negative control (isotonic NaCl). Increase of bile secretion was 180, 185, and 160%, in comparison to baseline (100%) after 15 min. Increase (135%) was also obtained with the ether extract of sandy everlasting applied in dose of 5 mg/100 g with a maximum after 30 min.

Szadowska (1962) also conducted experiments on antispasmodic activity on the smooth muscle isolated from rabbit and rat intestines and on gall-bladders isolated from guinea pigs and rabbits. Apigenin and the ether extract of *H. arenarium* that contains mainly apigenin, had the strongest antispasmodic activity on smooth muscles and isolated gall bladders *ex vivo*. Infusions and decoctions of *H. arenarium* flowers had weak spasmolytic activities.The drug is therefore mainly used as an adjuvant in the treatment of cholecystitis and cramp-like gallbladder disorders (Wichtl, 2001). In Europe, the therapeutic application includes treatment of various health issues such as cystitis, arthritis, rheumatism, and gout as well as for stimulating gastric secretion and for the treatment of gallbladder disorders (Shass, 1952; Vereschagin et al., 1959; Shikov et al., 2014).

Recently, Mao et al. (2017) reported anti-atherosclerotic activities of flavonoids (i.e., narirutin, naringin, eriodictyol, luteolin, galuteolin, astragalin, and kaempferol) isolated from the flowers of *H. arenarium*. They supposed that the main mechanism of the activity is through the pathway of anti-inflammation, especially by reduction of the expression of C-Reactive Protein (CRP), inhibition of the activities of the c-Jun NH_2_-terminal kinases (JNK2) and p38, and the mitogen-activated protein kinase (MAPK) pathway suppression.

Moreover, significant activities of naringenin, one of the main flavonoids of *H. arenarium*, were reported. Pafumi et al. (2017) found naringenin as the inhibitor of Two-Pore Channel 2 (TPC2) -mediated signaling, a key therapeutic step in a number of pathological conditions including the progression and metastatic potential of melanoma, Ebola virus infection, and Parkinson's disease. Park et al. (in press) suggest naringenin as a potential therapeutic molecule with anti-cancer effects on choriocarcinoma cells acting by inducing generation of ROS and activation of the MAPK pathways, while Liang et al. (2017) reported that naringenin protect keratinocytes from apoptosis and oxidative stress injury through inhibition of the NOD2-mediated NF-κB pathway. Furthermore, taking into account that naringenin and naringenin-5-*O*-glucoside are among the dominant compounds in *H. arenarium*, it is worth noted that Agus et al. (2017) reported that naringenin-rich fraction of pigeon pea leaves (*Cajanus cajan*) extract showed fairly well inhibitory effect toward *Salmonella thypi* in comparison with chloramphenicol.

## Cultivation approaches

Production of sandy everlasting for therapeutic use could be carried out by small farms as a niche product and since the sandy soil is needed, sandy terrains could be utilized for the production of this medicinal plant (Olsson et al., 2005). Although the sandy everlasting is very interesting for the pharmaceutical industry, and also classified as endangered species in a number of European countries, very few data about its cultivation are available in the literature. First attempts of growing *H. arenarium* date back to the mid of the 1970s (Fijalkowski and Seroczynska, 1974; Moroz et al., 1976; Pacholak and Zalecki, 1979; Sawilska et al., 2009), but none of them was completely successful in terms of surviving ratio and growth (Buchwald, 1992). Most of the authors concluded that this plant is inconvenient for cultivation since experimental trials yielded only slightly higher amounts of inflorescence biomass than those from natural stands. Tyszynska-Kownacka (1972) even suspected that this species might not be suitable for growing. Later cultivation trials have only explained the reason why those attempts failed (Sawilska, 2006, 2007, 2008). The main flaws of the attempts at growing sandy everlasting in the previous century were neglecting the clonal character of its growth and ignoring its mycorrhizal associations. Sawilska et al. (2009) took step forward in the further explanation on the necessity of mycorrhizal associations of arbuscular fungus *Glomus intraradices* with plant roots, but, although feasible, soil vaccination with mycorrhizal inoculum did not much influence the growth and flowering of single shoots. Sawilska and Jendrzejczak (2013) suggested *in vitro* method of propagation, which seems to be more profitable as it allows the acquisition of a potentially unlimited number of shapely plants that flower at the same time. Each method of cultivation proved to be feasible, as each yielded a particular amount of valuable, raw herbal material.

Similarly to the most of perennial species from Asteraceae family, the flowering biology of *H. arenarium* implies that the plant has to spend at least one year in rosette phenophase before it enters the generative phenophase (Pacholak and Zalecki, 1979; Sawilska et al., 2009). For the more detailed study of the species researchers tend to develop basic cultivation models with the use of agro-technical analysis methods, which could provide a study of the elements for industrial cultivation technology, in particular, to clarify the optimal methodology for seedlings production, time of planting, sowing rate, and maintains of established plantations.

The most comprehensive research that covers direct sowing and seedling production as plantation establishment methods has been published by Esmagambetova and Ahmetzanova (2006). In this work, authors reported that optimal plant density of 5–6 plants per 1 m^2^ was achieved by sowing rate of 2.5–3.0 kg/ha, and emphasized that although laboratory germination was 87%, the field of germination at sowing time was low, not exceeding 10.3%. Possible reason for this huge germination rate discrepancy could be in very small dimensions of *H. arenarium* achenes, where weak seed-soil contact could provoke germination without radicle rooting. Thousand seed weight (TSW) was estimated to about 0.06 g. Furthermore, observed field self-propagation by seeds in following 3 years of cultivation was very low (0.41%). Such a low realization of seed reproduction potential is common for wild-growing plants with small seeds, which have a high ratio of accidental fatality of seeds and seedlings associated with a limited supply of nutrients in the seed (Šohina and Valuckaya, 1984). This disadvantage could be overcome by increasing the sowing rate, which would consequently increase plant density and therewith competition between them, which leads to mutual inhibition of their development (Esmagambetova and Ahmetzanova, 2006). In the same report, authors suggested that mutual inhibition could be avoided after the thinning of plants in rows, which again leads to additional investments in both labor and material costs.

## Conclusions

*Helichrysi flos* (biological source *Helichrysum arenarium* (L.) Moench) is a well-known herbal drug in traditional medicine and it is used as a cholagogues, choleretic, diuretic, as mild spasmolytic, as a hepatoprotective agent and for detoxification. Recent studies pointed out significant biological activities of *H. areanarium* together with its main compounds such as flavonoids. On the other hand, there are no clinical data about testing the extracts or preparations based on *H. arenarium*. Although the sandy everlasting is classified as endangered species in a number of European countries and also very interesting for the pharmaceutical industry, none of the reported cultivation approaches proved to be successful. Additional efforts regarding cultivation methods, such as fertilization or inoculation with the range of mycorrhizal fungi, should be made to achieve sustainable agricultural production of this crop.

## Author contributions

DP took the lead in writing the manuscript and together with TJ conceived the main subject of this review. DP, TJ, DB, and KŠ wrote the Introduction section. DP summarized botanical description, taxonomy, and distribution of the species. TJ and DB contributed to chemical constituents of the species, while KŠ and SJ summarized reported knowledge about traditional uses and cultivation approaches of the species.

### Conflict of interest statement

The authors declare that the research was conducted in the absence of any commercial or financial relationships that could be construed as a potential conflict of interest.
